# Global prototype distillation for heterogeneous federated learning

**DOI:** 10.1038/s41598-024-62908-0

**Published:** 2024-05-27

**Authors:** Shu Wu, Jindou Chen, Xueli Nie, Yong Wang, Xiancun Zhou, Linlin Lu, Wei Peng, Yao Nie, Waseef Menhaj

**Affiliations:** 1 School of Electronic and Information Engineering, West Anhui University, Lu’an, 237012 China; 2 School of Physics and Electronic Information, Anhui Normal University, Wuhu, 241002 China; 3 MoE Key of Artificial Intelligence, AI Institute, Shanghai Jiao Tong University, Shanghai, 200240 China; 4 School of Computer Engineering, Baluchistan University of Information Technology Engineering and Management Sciences (BUITEMS), Quetta, 87300 Pakistan

**Keywords:** Federated learning, Knowledge distillation, Data heterogeneity, Information technology, Software

## Abstract

Federated learning is a distributed machine learning paradigm where the goal is to collaboratively train a high quality global model while private training data remains local over distributed clients. However, heterogenous data distribution over clients is severely challenging for federated learning system, which severely damage the quality of model. In order to address this challenge, we propose global prototype distillation (FedGPD) for heterogenous federated learning to improve performance of global model. The intuition is to use global class prototypes as knowledge to instruct local training on client side. Eventually, local objectives will be consistent with the global optima so that FedGPD learns an improved global model. Experiments show that FedGPD outperforms previous state-of-art methods by 0.22% ~1.28% in terms of average accuracy on representative benchmark datasets.

## Introduction

Federated Learning (FL), as an effective machine learning paradigm for decentralized data, has enabled distributed clients to collaboratively train a global model. In Federated Learning setting, the clients independently train local models based on their own dataset on each round. The trained local models’ parameters will be sent to a server for aggregation to produce a global model without accessing their private local data. Serving as a communication-efficient and privacy-preserving learning scheme without sharing clients’ private data, FL has shown its potential to facilitate real-world applications, such as healthcare analysis^1,2^, biometrics analysis^3^, and object detection, etc^4,5^.

FedAVG^6^, as a classical FL algorithm, achieves an aggregated global model by taking a weighted average based on data quantity of each client. Although it has been proven superior on the independent and identically distributed (IID) data, the serious challenge of FL in practice is that clients’ data are usually heterogenous. In other words, each client’s local dataset is non-iid distributed. A drift of local model training caused by the unbalanced data distribution will make the local objective of each client far away from the global optima, which results in a significant degradation of FL system performance^7^. Therefore, simply performing element-wise averaging of model parameters, as most FL algorithms adopt, would not produce an ideal global model to serve all clients^8^. In addition, some related schemes have been used in federated learning, e.g., data poisoning attack^9^ and federated class-incremental learning^10,11^ improve on the original federation averaging algorithm, respectively. But they mainly address the problem of model training in specific federated learning scenarios. They do not deal much with the data heterogeneity problem in federation learning.

Furthermore, a variety of researches have attempted to tackle data heterogeneity problem, which mainly contain two complementary perspectives: one focuses on effective and accurate model aggregation, such as FedNova^12^, FedMA^13^. Another aims to prevent local training from overfitting biased local dataset on client side, such as MOON^14^, FedProx^15^. Most of methods try to regulate the local model drift from global model parameter perspective. However, these approaches lack exploration of input data underlying information. Knowledge distillation is also introduced into FL to solve model aggregation problem. Based on a proxy dataset, knowledge distillation can reduce model drift problem via instructing global model with the ensemble local models^16–18^. However, the existence of a proxy dataset may not always be realizable in practice. To overcome the above challenge of data heterogeneity, this work presents a novel global prototype distillation method, named FedGPD.

The motivation of the algorithm is coming from a common observation in deep learning: the model trained on the overall dataset can extract a better feature representation than the model trained on the biased sub-dataset. For example, the model trained on cat and dog images can extract much more cat’s features than the model only trained on dog images. Apparently, in FL setting, the model trained on skewed local dataset would learn poor feature representations. As a consequence, each client’s inconsistent local training will degrade the performance of the whole system. However, global model can be considered as a more powerful model trained on distributed data. Therefore, global model would extract more effective representations than local models. Based on above considerations, utilizing global knowledge from global model to correct each client’s local training is the key to solving heterogenous data distribution. The intuition is that with the help of global knowledge, pulling the clients’ local drift towards the corresponding global model is a feasible method, thus making the local training consistent with the global objective. Hence, the non-IID problem would be tackled.

Prototype learning^19^ is introduced into FL to learn an effective global representation by fully using global model information. A prototype is calculated as the mean of the feature vectors within each class, which is a good representation of input data on corresponding class. Therefore, every client can compute class prototypes on its own dataset to represent local information. During communication period, clients not only send model parameters but also local prototypes to servers for aggregation. After gathering the class prototypes from each client, the server can calculate the global prototype. It can be regarded as the global knowledge and delivered back to clients. After receiving global prototype, clients use the proposed local objective to regulate the local training. With the assistance of knowledge distillation^20^, client uses the global prototype on its own classes as transferred knowledge to rectify local loss function, in order to achieve consistence with the global optima. The advantage of knowledge distillation on client side is to avoid the problem of public proxy dataset.

**Figure 1:**
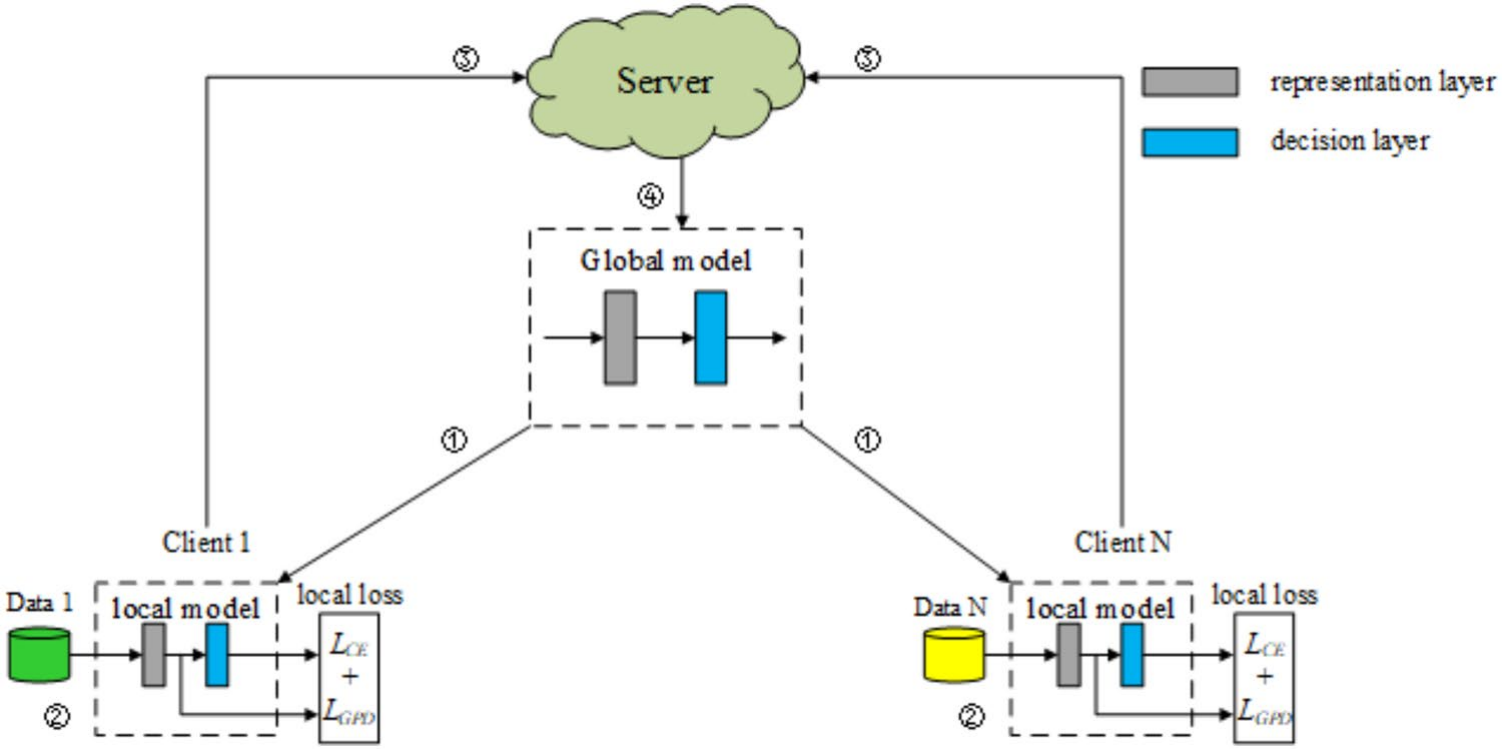
Overall architecture of FedGPD.

FedGPD promotes each instance of local dataset to approach the global prototype of its corresponding class. Hence, the performance of local model would be improved. It tackles heterogeneity problem from a new combination between prototype learning and knowledge distillation. The overall architecture is shown in Fig. 1. The specific steps can be described as follows: Firstly, the server creates a global model and sends all of its parameters to every local client. Secondly, the local terminal is used to train the client to obtain the parameters of the local model. Thirdly, the local client transmits global model parameters $w^t$ and global prototype $\mathbb {C}_k$ to the server. Finally, after obtaining these two parameters, the server aggregates global model parameters $w^t$ and global prototype $\mathbb {C}_k$ respectively. Meanwhile, the server sends the updated global model $w^{t+1}$ to each local client again.

**Contributions**:

1. We propose a novel federated learning algorithm called FedGPD. In order to address data heterogeneous problem, the global prototype is introduced as distilled knowledge to regulate local training and to avoid public proxy dataset problem.
2. We devise a global prototype distillation loss function to take advantage of the underlying representation of global prototype. Conventional distillation method is improved. Instead of teacher model’s logistic, utilizing aggregated global prototypes brings a good performance of FedGPD.
3. We implement exhausted experiments to evaluate the performance of proposed algorithm on different public image classification datasets, including MNIST, CIFAR-10 and CIFAR-100. FedGPD outperforms the state-of-the-art federated learning algorithm only by a lightweight modification on local loss function.

## Background and related work

### Federated Learning with statistical heterogeneity

Federated Learning (FL) is a distributed learning framework, where a group of local clients conduct local training task and a central server coordinate the aggregation process. It eventually leads to an effective aggregated global model. Users’ raw data are kept locally and only model parameters are sent to server so that the data privacy will be preserved. Therefore, FL is an effective and secure machine learning paradigm for decentralization of data sources.

As the first representative algorithm, FedAVG is a de facto optimization method to achieve an aggregated model by averaging the local models’ parameters from distributed local clients. Typically, in each round of FedAVG, there are four steps: (1) Server initializes a random global model and sends the parameters to each client who participates in training. (2) Each client updates the local model according to their local data after receiving the global model. (3) Clients upload the local model parameters to server for aggregation. (4) Server takes weighted mean of all received parameters based on data quantity of each client to produce a global model for next training round. These four steps will stop until achieving convergence or reaching maximum communication rounds.

Suppose there are *N* clients. Client *i* holds a local dataset $D_i$ . The general goal of FL is to learn a global model weight *w* over the dataset $\mathbb {D}=\bigcup \left\{ D_{i}\right\} _{i=1}^{N}$, while the clients’ raw data will never be communicated with others. The objective of FL is to optimize:

1 $$\begin{aligned} \min _{w} L(w)=\sum _{i=1}^{N} \frac{\left| D_{i}\right| }{|\mathbb {D}|} L_{i}(w) \end{aligned}$$

where $L_{i}(w)=\mathbb {E}_{(x, y) \sim D_{i}}\left[ l_{i}\left( F_{i}(w ; x), y\right) \right]$ is local objective function for client *i*, $F_{i}(w; x)$ represents the local model and $l_{i}(\cdot , \cdot )$ is the loss function.

On the independent and identically distributed (IID) data setting, FL achieves superior performance. However, users’ data from real-world is often non-iid distributed which causes heavy damage to the aggregated model. Therefore, statistical heterogeneity across clients is the most significant challenge for FL. There are quite some researches focusing on tackling non-iid problem in FL, which are mainly divided into two perspectives: (1) improve the aggregation algorithm, such as FedNova, FedMA; (2) stabilize the efficacy of local training, such as MOON, FedProx.

For global model aggregation, FedMA uses Bayesian non-parametric methods to compare and takes weighted mean in a layer-wise way. FedNova normalizes the local updates before taking average. In^21^, q-Fair Federated Learning enables all participants to maximize the performance of their local model under a certain global model by modifying the aggregation algorithm. FedAvgM^22^ proposes a mitigation strategy for the Federated Averaging algorithm via server momentum. FedMIX^23^ utilizes Mean Augmented Federated Learning (MAFL), where clients send and receive averaged local data, to improve performance. FedBE^24^ takes a Bayesian inference perspective to ensemble global models, leading to much robust aggregation. MHAT^25^ introduces a novel model-heterogenous aggregation training federated learning scheme to extract the update information of the heterogenous model of all clients. Fortunately, FedGPD is orthogonal to above methods. The combination of these works is promising because FedGPD focuses on local training phase instead of model aggregation.

For stabilization of local training, FedProx proposes a proximal term to optimize local objective. The distance between global model and local model is computed to restrict local model parameters to approach global optimal instead of overfitting local dataset. SCAFFOLD^26^ introduces control variates to correct the drift in local training phase. MOON takes advantage of model-level contrastive learning to decrease the distance between global model representation and local model representation. It increases the distance between local model representation and previous local model representation.

### Knowledge distillation

Knowledge Distillation (KD) is a novel technique to transfer knowledge from a complex but effective teacher model $w^T$ to a lightweight but less effective student model $w^S$^27–29^. It keeps student model as light as possible without losing performance. The intuition of KD is to minimize the divergence between logits outputs from teacher model and student model. Therefore, student model can learn the knowledge from teacher model and acquire a better performance because student model could approach output from teacher model^30,31^. The loss of knowledge distillation could be computed by Kullback-Leiler divergence:

2 $$\begin{aligned} \min _{w^{S}} K L\left( \sigma \left( F\left( w^{T}; x\right) \right) , \sigma \left( F\left( w^{S}; x\right) \right) \right) \end{aligned}$$

Based on the type of transferred knowledge, knowledge distillation can be divided into three categories: (1) output transfer; (2) feature transfer; (3) relation transfer. FedGPD belongs to the second category. The global model prototype is taken as knowledge to correct local training.

Moreover, the concept of KD has been brought into FL to deal with heterogeneity problem. FD^32^ synchronizes logits per label which are accumulated during the local training. The averaged logits per label (over local steps and clients) will then be used as a distillation regularizer for the next round’s local training. FedDF^33^ treats local models as teacher models and evaluates each model on unlabeled public data stored on server side. Then, average logit outputs of teacher models are used as knowledge to train global student model. FedBE introduces Bayesian model ensemble into FL so that the ensemble predictions as the pseudo-labels of public unlabeled data are considered as teacher to train a student global model. These methods usually treat local models as teachers. Then their knowledge will be transferred to a student global model. FedAUX^34^ improves performance by deriving maximum utility from the unlabeled auxiliary data, where the ensemble predictions on the auxiliary data are weighted according to the certainty of each client model.

The huge drawback of these method is that they all need a public dataset or a proxy unlabeled dataset to complete knowledge distillation. In order to address the above problems, FedGPD uses global prototype as transferred knowledge and knowledge distillation happens on client side. Thus, there is no requirement of any proxy dataset and additional training on server side.

## Proposed global prototype distillation Federated learning

**Figure 2:**
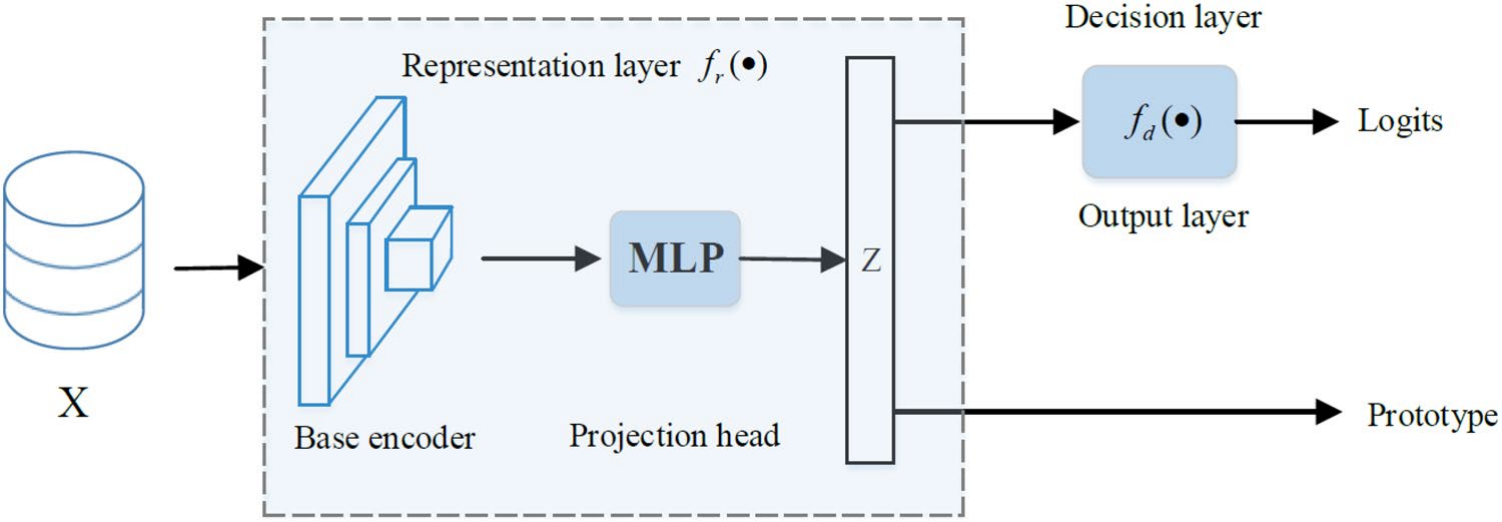
Overview of the local network architecture.

In this section, a summary of whole proposed algorithm is shown in Algorithm 1. An overview of its learning procedure is illustrated in Fig. 2. The general flow can be described as follows: Firstly, the data set *X* is fed into the neural network of the local client and projected by the MLP as *z*. Secondly, the neural network mapping result for the local client is logits. Then it is used for Softmax to compute classification probabilities. Finally, the local prototype obtained from the local client will be uploaded to the server once more.

### Problem formulation

The considered heterogeneous FL problem is formulated as follows. Suppose there are *N* clients in the system. Each client possesses a labeled image dataset $D_i:=\{(x_{m}^{i},y_{m}^{i})\}_{m=1}^{|D_i|}$, $i=1,...,N$, where $x_{m}^{i}$ is the *m*-th data sample in *i*-th client. $y_{m}^{i}\in \{1,2,...,K\}$ is the corresponding label among *K* classes, and $|D_i|$ denotes the number of training samples owned by the *i*-th client. The datasets of different clients may be drawn from different distributions $p_{i}$, $i=1,...,N$. Dirichlet distribution $Dir_{N}(\beta )$ is used to simulate label distribution skew among clients, which is most common federated setting. The goal is to train models for the image classification task among the clients. In other words, under non-iid setting where clients’ data distribution $p_{i}$ are quite different from each other, there is a requirement to optimize $L(w)=\sum _{i=1}^{N} \frac{\left| D_{i}\right| }{|\mathbb {D}|} L_{i}(w)$.

**Algorithm 1:**
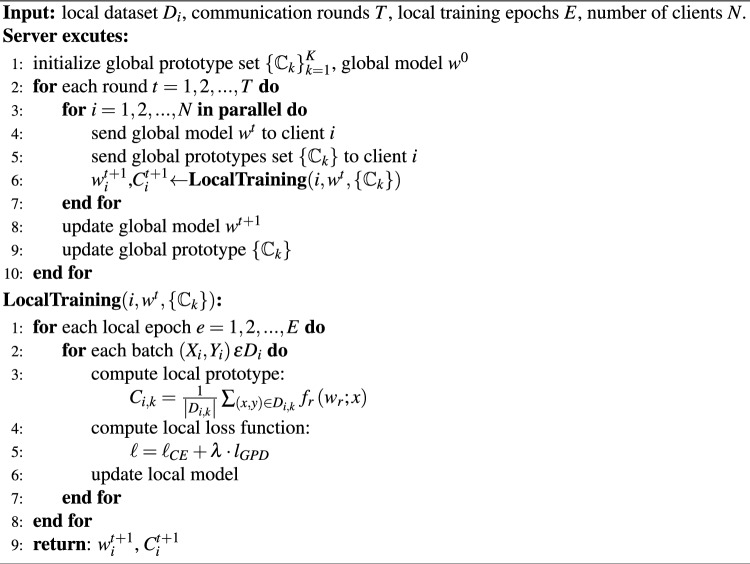
FedGPD

### Architecture of FedPGD

$\bullet$ **Client local network design**

A typical neural network generally consists of three components: the base encoder extracts representation from inputs; the project header is used to map the representation to a new feature space; the output layer produces the classification decisions for each class. Furthermore, the base encoder and project header form representation layer; output layer is actually decision layer^14^. **Representation layer** maps the input data *x* from the original feature space to representation space. The extracting and mapping function is denoted by $z=f_{r}\left( w_{r}; x\right)$, where *z* is mapped representation of *x* and $w_r$ is learnable parameters of representation layer. **Decision layer** makes the classification decision for specific learning tasks which generates a prediction from mapped representation *z*. $s=f_{d}\left( w_{d}; z\right)$ is to denote the prediction function, where *s* is output prediction and $w_d$ is learnable parameters of decision layer. Therefore, a complete network could be denoted as:

3 $$\begin{aligned} F(w; x)=f_{d}\left( w_{d}; f_{r}\left( w_{r}; x\right) \right) \end{aligned}$$

**Prototype** is a proxy of one class in classification tasks. It can be measured as the mean value of the feature vectors in every class. For the *i*-th client, the local prototype $C_{i,k}$ is the mean representation of inputs in class *k*.

4 $$\begin{aligned} C_{i, k}=\frac{1}{\left| D_{i, k}\right| } \sum _{(x, y) \in D_{i, k}} f_{r}\left( w_{r}; x\right) \end{aligned}$$

where $D_{i,k}$ is the subset of local dataset which consists of training data belonging to class *k* in client *i*.

Figure 2 represents the local network architecture. Notice that there is the similar network architecture as FedProto^35^, but this works uses different methods to solve non-iid problem. Logits output is used for conventional cross entropy loss. Besides, local prototype from representation layer is considered as an input of distillation term on local loss function. The design of the prototype regularization term in the local training loss function is different from FedProto.

$\bullet$ **Whole algorithm design**

The intuition of FedGPD is from knowledge distillation and prototype learning. Under non-iid setting, local model would overfit its own biased local dataset. It results in a significant degradation of global model’s effectiveness. However, aggregated global model is supposed to acquire a better performance than each local model. Global model has a superior ability to extract information from decentralized global dataset. Therefore, the global model could be considered as a teacher model to instruct each local model as a student model to avoid overfitting its own biased local dataset. However, directly using the logits from global model as knowledge could lose partial inputs feature information. Considering prototype is the representation of input data which means it contains more feature information, global prototype, as the representation of global dataset, is employed to instruct local model to better fit the whole data distribution. Clients will upload local prototypes together with model parameters to server for aggregation.

**Global prototype** after gathering local prototypes from *n* clients, the aggregated global prototype is computed by averaging them. $\mathbb {C}_k$ is global prototype in class *k*, which is weighted mean of local prototypes corresponding to class *k*. Therefore, the overall global prototype set is $\left\{ \mathbb {C}_{k}\right\} _{k=1}^{K}$, where *K* is total numbers of labels.

For better explanation, FedGPD is described in Algorithm 1. There are four main steps: (1) Server initializes a random global model and sends the parameters to each client who participates in training. (2) each client updates the local model according to proposed loss function after receiving the global model. (3) clients upload the local model parameters and local prototypes to server for aggregation. (4) server aggregates local model parameters and local prototypes to produce a global model and global prototype respectively for next round. These four steps will stop until achieving convergence or reaching maximum communication rounds.

### Global prototype distillation

**Local Objective**: the designed local loss function is comprised of two parts. The first part is a typical cross-entropy loss term $\ell _{CE}$ pervasively used in supervised learning task. The second part is proposed global prototype distillation loss term denoted as $\ell _{PGD}$. This term makes the local model learn global information from representation layer so that the local prototypes will approach global prototypes. This work introduces hyperparameter $\lambda$ to control the weight of $\ell _{PGD}$.The final loss function for the local model is:

5 $$\begin{aligned} \ell =\ell _{C E}+\lambda \cdot l_{G P D} \end{aligned}$$

**Global prototype distillation loss**: in order to mitigate over-biased local model, this work brings knowledge distillation into local training phase. Utilizing the global prototype as teaching information, FedGPD formulates the distillation loss as:

6 $$\begin{aligned} \ell _{G P D}=-\sum _{k}^{K_{i}} \mathbb {C}_{k}^{T} \log \left( C_{k}^{T}\right) \end{aligned}$$

where $\mathbb {C}_{k}^{T}$ is softmax output of global prototype on temperature *T* in class *k*, $C_{k}^{T}$ is softmax output of local prototype on temperature *T* in class *k* and $K_{i}$ is the total number of classes in client *i*.

## Experiments

### Experimental setup

Experiments are conducted to mainly compare FedGPD with three state-of-art federated learning algorithms, including (1) FedAvg, (2) FedProx, (3) MOON,and SOLO, where each client trains its own model on local dataset without federated learning. Experiments datasets are three common open source datasets: MNIST, CIFAR-10, CIFAR-100. To be fair, the same model are used in local training for all algorithms. Two different neural networks are applied: (1) a simple CNN model plus a fully connected layer as representation layer for MNIST and CIFAR-10. (2) ResNet-18 plus a fully connected layer as representation layer for CIFAR-100. Specifically, the simple CNN model contains two 5*5 convolution layers followed by 2*2 max pooling and two fully connected layers with ReLU activation.

The output dimension of the representation layer decides the size of prototype, which affects the following knowledge distillation. Therefore, the output dimension is set as a hyperparameter under tuning. By default, 256 is a common choice followed by other algorithms for a fair comparison.

This work uses PyTorch to implement the algorithm and the other baselines. SGD optimizer with learning rate 0.01 is set for all algorithms. SGD weight decay and momentum are set to 0.00001 and 0.9 respectively. Training batch size is set to 64. The number of local training epochs is set to 10 by default and the number of communication rounds is set to 50 for MNIST and 100 for CIFAR-10/CIFAR-100. Distillation temperature *T* and global prototype distillation loss weight $\lambda$ are tunable hyperparameters and are set $T=2$, $\lambda =0.05$ respectively for default.

Following the previous works, Dirichlet distribution is utilized to generate the non-IID distribution among clients. Concretely, sample $p_{k}\sim {Dir}_{N}(\beta )$ and allocate a $p_{k,i}$ proportion of the instances of class *k* to client *i*, where $\beta$ is a concentration parameter controlling the degree of non-IID distribution. The default configuration is list on Table 1.

**Table 1:** The default configuration.

Parameter	Default setting
Learning rate	0.01
Batch size	64
Number of clients	10
Number of communication rounds	100
Number of local epochs	10
Concentration Parameter	0.1
Sample fraction	1.0
Out dim	256

### Accuracy results

Table 2 lists the test accuracy of FedGPD and other baselines. Under heterogeneous setting, SOLO shows the worst result among all the methods, which proves the benefits of federated learning. FedAVG, as a basic algorithm for FL, uses cross-entropy to train the local models and weighted average to aggregate model parameters. But it achieves relatively low accuracy under non-IID setting. Moreover, FedProx makes little modification on the FedAVG by adding a proximal term on loss function. As a result, its accuracy is very close to FedAVG, especially when parameter $\mu$ is small.

**Table 2:** Test accuracy of FedGPD and the other methods on test dataset.

Method	MNIST (%)	CIFAR-10 (%)	CIFAR-100 (%)
Solo	66.31	46.30	22.30
FedAVG	66.31	46.30	22.30
FedProx	97.07	62.93	63.70
MOON	98.20	63.50	63.92
FedGPD	98.52	64.78	64.22

**Figure 3:**
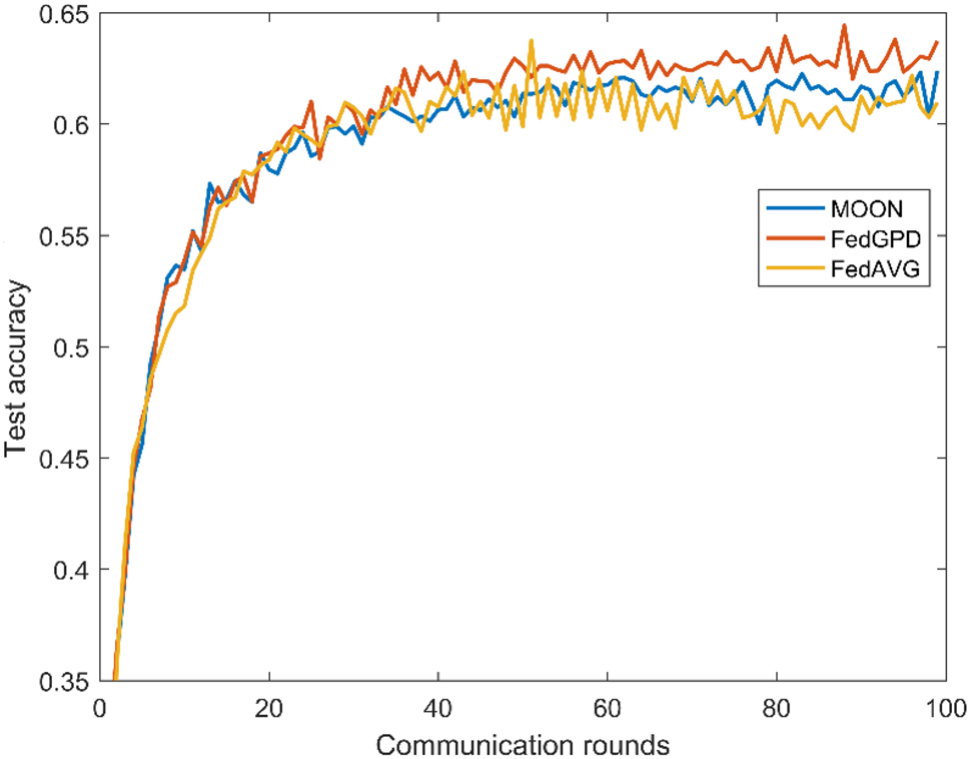
Test accuracy with different number of communication rounds on CIFAR10.

**Figure 4:**
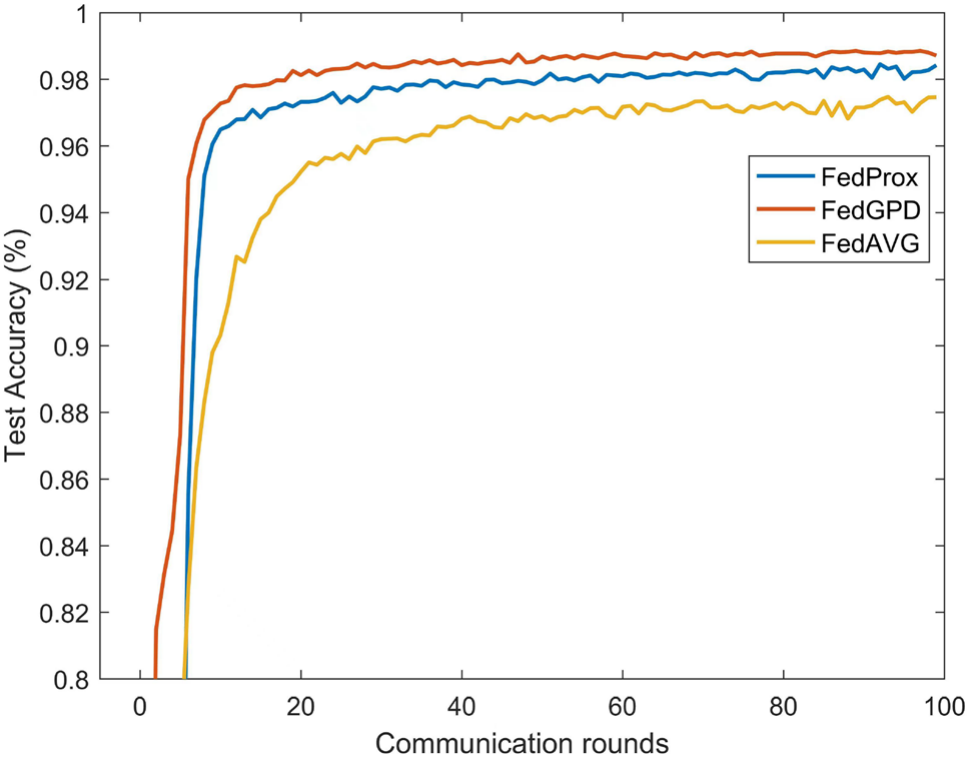
Test accuracy with different number of communication rounds on MNIST.

Furthermore, MOON proposes a model-level contrastive federated learning with model-contrastive loss to deal with heterogeneous problem. MOON achieves great performance on slightly heterogeneous setting and even better than FedGPD under the condition of $\beta =1$. However concentration parameter $\beta$ is 0.1, which means non-iid problem is severe and is more consistent with the practical situation, FedGPD provides better results. According to Figs. 3 and 4, FedGPD outperforms FedProx on MNIST dataset and also acquires higher test accuracy than MOON on CIFAR10 dataset. Therefore, FedGPD achieves competitive or even better performance than other methods on the different datasets. It demonstrates that FedGPD can effectively rectify the local training by using global prototype distillation.

#### Comparison with logits distillation method

The concept of original KD is to transfer the knowledge via minimizing the KL-Divergence between prediction logits of teachers and students. while this work chooses feature-based knowledge distillation instead of logits-base knowledge distillation. To demonstrate the superiority of global prototype distillation method, two additional distillation methods are designed for comparison:

First method is directly to use the previous round global model logits output as distilled knowledge, denoted as **method 1**. Specifically, distillation loss in method 1 is computed as: $\ell _{method1}={\varvec{KL}}({\varvec{\bar{p}||q}})$, where ${\varvec{\bar{p}}}=[\bar{p}_1,\bar{p}_2,...,\bar{p}_K]$. $\bar{p}_k$ is computed by

$\frac{exp(z_k)}{\sum _{j=1}^{K}exp(z_j)}$, where $z_k$ represents the logit of the *k*-th class from previous global model. ${\varvec{{q}}}$ is logits from local model.

Second method is to aggregate local model logits to form global logits as distilled knowledge, denoted as **method 2**. Specifically, distillation loss in method 2 is computed as:$\ell _{method2}={\varvec{KL}}({\varvec{\hat{p}||q}})$, where ${\varvec{\hat{p}}}=[\hat{p}_1,\hat{p}_2,...,\hat{p}_K]$. $\hat{p}_k$ is computed by average all local model logits for *k*-th class. ${\varvec{{q}}}$ is logits from local model.

Table 3 lists the test accuracy of FedGPD and other baselines. It can be seen that the accuracy of our FedGPD is 0.27% ~3.08% higher than the other two comparison schemes. FedGPD performs better than other two distillation methods because global prototypes extract more useful information than logits output. Therefore, the novel global prototype distillation method of FedGPD transfers more effective instruction knowledge to rectify local training.

**Table 3:** Test accuracy comparison with other KD methods.

Method	MNIST (%)	CIFAR-10 (%)	CIFAR-100 (%)
Method 1	98.32	61.70	63.08
Method 2	98.25	62.57	63.17
FedGPD	98.52	64.78	64.22

#### Influence of number of communication rounds

Figures 3 and 4 show the test accuracy in each communication round during training on different dataset. FedGPD achieves the best performance at the end of the training. It is worth noting that FedGPD achieves the same accuracy faster than other algorithms. Therefore, FedGPD is more communication-efficient than other approaches.

#### Influence of number of local epochs

Through the study of influence of number of local epochs (E), it is found that there is a trade-off between local epochs and model performance. When E is set to 1, the local update is very small so the local network cannot be well trained. The test accuracy is relatively low. However, when E is too large, local model would overfit the skewed local dataset, which leads to a degradation of test accuracy. Table 4 shows that number of local epochs is set to 10, which turns out a suitable choice.

**Table 4:** Test accuracy of FedGPD with different number of local epochs.

Local epochs	1	2	3	4	5
FedGPD	59.10%	62.60%	**64.78%**	60.27%	56.23%

Significant values are in bold.

#### Influence of data heterogeneity

It is important to investigate the influence of data heterogeneity by changing the concentration parameter $\beta$ of Dirichlet distribution to evaluate the performance of algorithms. For a smaller $\beta$, the data distribution will be more skewed. The results are listed on Table 5. Although MOON gets better test accuracy when $\beta =1$, FedGPD achieves better performance when $\beta =0.1$ or 0.5. This demonstrates that global prototype distillation is more effective under extremely heterogenous condition.

**Table 5:** Test accuracy of FedGPD and the other methods on test dataset.

Method	$\beta =0.1$ (%)	$\beta =0.5$ (%)	$\beta =1$ (%)
FedAVG	62.90	68.50	70.35
FedProx	62.93	69.20	70.69
MOON	63.50	70.22	**72.23**
FedGPD	**64.78**	**70.53**	71.32

Significant values are in bold.

#### Influence of coefficient in loss function

**Figure 5:**
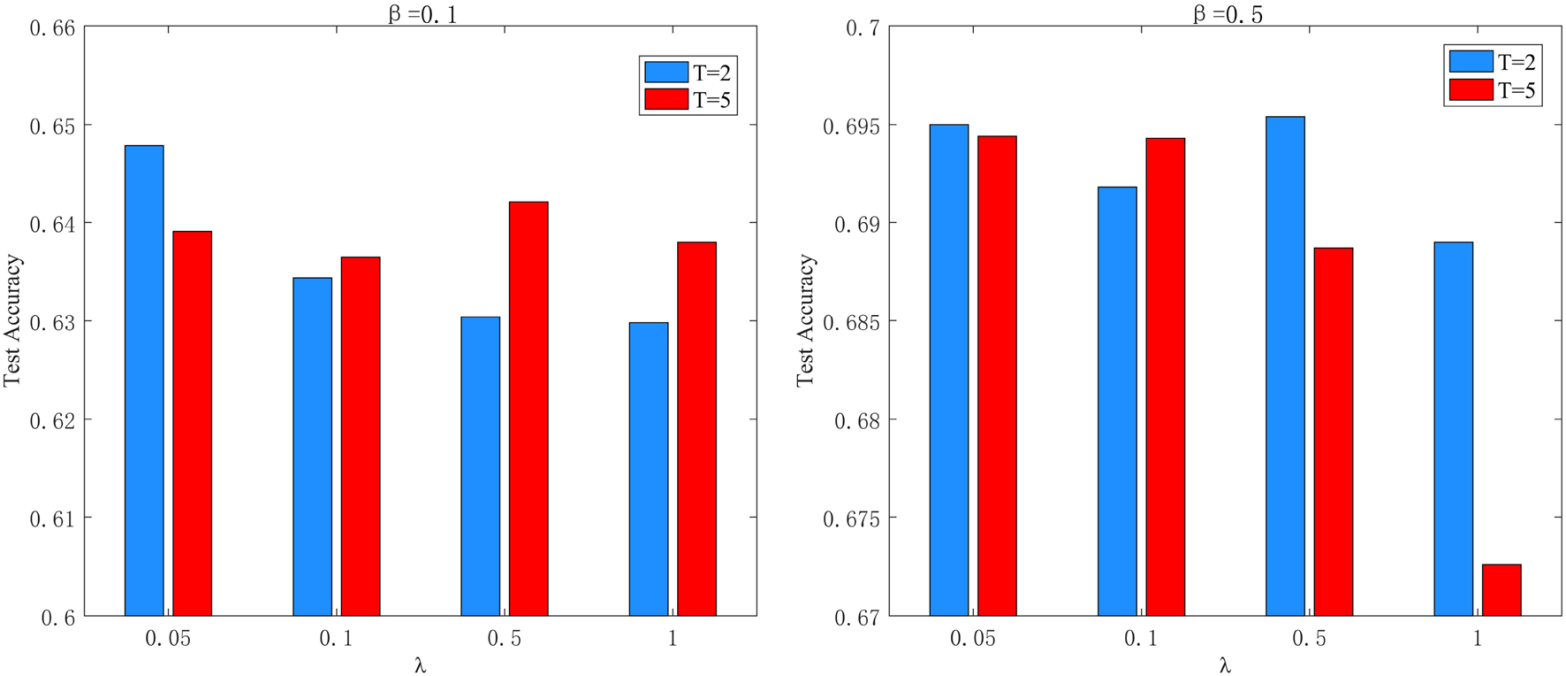
Coefficients comparison under different $\beta$.

The two important coefficients in $\ell _{G P D}$ are distillation temperature *T* and weight hyperparameter $\lambda$. Distillation temperature *T* controls the importance of each soft target. As the temperature increases, the distribution of soft target will be smoother. Weight hyperparameter $\lambda$ controls the proportion of $\ell _{G P D}$ in whole loss function. This works tries *T* from 2, 5 and $\lambda$ from 0.05, 0.1, 0.5, 1 on CIFAR10 dataset, shown on the Table 6. It has been found out that $T=2$ is a common choice even when $\beta$ is 0.1. But for weight hyperparameter $\lambda$, when $\beta$ is 0.5, the choice of $\lambda$ slightly influences test accuracy especially $\lambda =0.05$ or 0.5. Therefore, according to Fig. 5, $T=2$ and $\lambda =0.05$ are set as the default values.

**Table 6:** Test accuracy of *T* from {2, 5} and $\lambda$ from {0.05, 0.1, 0.5, 1} on CIFAR10 dataset with different $\beta$.

Parameters	$\beta = 0.1$		$\beta = 0.5$	
	$T = 2$ (%)	$T = 3$ (%)	T = 2 (%)	$T = 5$ (%)
$\lambda =0.05$	**64.78**	$63.91$	69.50	69.44
$\lambda =0.1$	63.44	63.65	69.18	69.43
$\lambda =0.5$	63.04	64.21	**69.54**	68.87
$\lambda =1.0$	62.98	63.80	68.90	67.26

Significant values are in bold.

#### Comparison with FedProto

FedProto conducts MSE to measure the distance between local prototype and global prototype, and uses *n*-way *k*-shoot to divide datastet. Therefore, not only data classes of train dataset and test dataset are the same on one client, but also there is no global model to aggregate, where local models are more personalized and whole settings are totally different. If directly using FedProto into Dirichlet distribution setting, local model won’t be able to gain ability of global model to deal with the whole test dataset because each local model is tested on its own distributed partial test dataset. Experiments with *n*-way *k*-shoot could not simulate the longtail problem in reality. However, for fair comparison with FedProto, following their experiment setting (dividing dataset by 3-way 100-shoot), global prototype distillation is used to rectify local model.

Experiments evaluate local model performance instead of global model. Results are shown in the Table 7. FedGPD achieves equivalent and even better performance. Although there is a global model to aggregate model parameters, the effectiveness of FedGPD has been proven. With correction term of Global prototype distillation, local models can effectively handle its respective heterogeneous datasets, and the global model also performs well on the entire test dataset.

**Table 7:** Average test accuracy of local models compared with FedProto.

Method	MNIST		CIFAR-10	
	$Stdev = 2$ (%)	$Stdev = 3$ (%)	Stdev = 1 (%)	$Stdev = 2$ (%)
FedGPD	98.62	$98.05$	75.01	68.24
FedProto	98.67	97.61	75.18	67.47

### Communication cost and limitation

Communication costs are always a challenge in FL. In general, communication costs in most FL algorithms are decided by size of shared model parameters. For instance, in FedAVG, clients and server have the same network architecture. Therefore, size of model parameter will affect the communication cost. Take CIFAR10 as an example, simple CNN model in this work contains two 5*5 convolution layers followed by 2*2 max pooling and two fully connected layers with ReLU activation. The total number of parameters is 85486. In FedGPD, except for model parameters, prototypes are needed to be transmitted between server and clients. However, compared to model parameters, the size of prototype is relatively small. Default prototype size for each class is 256. The extra communication cost is 2560 at most, which is negligible. Therefore, the proposed global prototype distillation method can outperform most of the state-of-the-art FL schemes within a slight communication overhead.

One possible limitation is that sending local prototypes as well as model parameters would put local dataset in the threat of privacy leakage. However the focus of this work is dealing with heterogenous data distribution problem in FL. Local prototype can be transmitted via lightweight encryption because prototype size of each class is 256, which is relatively small compared to model parameters. Therefore, security issue can totally be handled by applying some lightweight encryption algorithm.

### Ethical approval

This article does not contain any studies with human participants or animals performed by any of the authors.

## Conclusion

This work presents FedGPD, a novel global knowledge distillation method to use the global class prototypes to rectify the local objective function in order to mitigate the local bias problem. Particularly, prototype is a proxy of one class in classification tasks, which contains the enormous information of input instances. Therefore, global class prototypes are an effective knowledge to instruct local training. Extensive experiments are conducted on MNIST, CIFAR-10/100 datasets under the setting of non-IID. Results show that FedGPD achieves comparable or better results within a slight communication overhead.

## Data Availability

The data required for reproducing the results in this work is available at the following URL: MNIST: http://yann.lecun.com/exdb/mnist/.
